# Characteristics of dysarthria in patients with spinocerebellar degeneration and multiple system atrophy: a cross-sectional and longitudinal study using the Frenchay Dysarthria Assessment Second Edition (FDA-2)

**DOI:** 10.3389/fneur.2025.1734012

**Published:** 2026-01-07

**Authors:** Arisa Kawabata, Ayako Wada, Michiyuki Kawakami, Kentaro Kaji, Kenta Nakanishi, Tomoyoshi Otsuka, Tetsuya Tsuji

**Affiliations:** 1 Department of Rehabilitation Medicine, National Hospital Organization Higashisaitama National Hospital, Saitama, Japan; 2 Department of Rehabilitation Medicine, Keio University School of Medicine, Tokyo, Japan

**Keywords:** dysarthria assessment, FDA-2, multiple system atrophy, speech impairment, spinocerebellar degeneration

## Abstract

**Purpose:**

The purpose of this study was to evaluate the dysarthria of patients with spinocerebellar degeneration and multiple system atrophy (SCD/MSA) cross-sectionally and longitudinally using the Frenchay Dysarthria Assessment Second Edition (FDA-2), which provides a comprehensive assessment of dysarthria, and to clarify characteristic patterns and longitudinal changes.

**Materials and methods:**

A total of 21 patients with SCD and MSA were included. Patients’ dysarthria was assessed using the FDA-2 and the Scale for the Assessment and Rating of Ataxia (SARA) item 4(SARA speech) at intervals of ≥ 12 months. Changes over time were examined with a paired t-test and Wilcoxon’s signed-rank test. Multiple regression analysis explored determinants of speech intelligibility, with FDA-2 intelligibility as the dependent variable and respiration, lips, palate, laryngeal, and tongue subscores as predictors.

**Results:**

The number of patients according to disease types was SCA2 in 1, SCA3 in 8, SCA31 in 1, MSA-C in 8, and an undetermined disease type in 3. The examination interval was 31.1 ± 15.6 months. FDA-2 scores were lower for repetitive-movement or speech items than for rest items. Lips and palate subscores were relatively higher, whereas laryngeal and tongue subscores were lower. Over time, reflex, lips, laryngeal, tongue, and total scores declined significantly, whereas the palate, respiration, and intelligibility scores remained stable. Multiple regression identified the laryngeal subscores as the only significant predictor of intelligibility (Sβ = 0.77, *p* = 0.047).

**Conclusion:**

The FDA-2 revealed a distinct dysarthria profile in SCD/MSA. Longitudinal FDA-2 monitoring may facilitate early detection and targeted intervention.

## 1 Introduction

Spinocerebellar degeneration (SCD) is a progressive neurodegenerative disease that affects the cerebellum, brainstem, spinal cord, and cerebrum, causing a variety of symptoms including cerebellar ataxia, parkinsonism, pyramidal tract disorders, peripheral neuropathy, and cognitive decline (1). Spinocerebellar ataxias (SCA) represent a heterogeneous group of autosomal dominant disorders within the spectrum of SCD. Multiple system atrophy (MSA), which is generally considered a sporadic disease and defined separately from SCD, presents with cerebellar ataxia, parkinsonism, and autonomic dysfunction; as the disease progresses, lesions extend to additional systems, and clinical syndromes overlap (2). Cerebellar ataxia is a core clinical manifestation of SCD and MSA, resulting in impaired motor coordination, muscle hypotonia, and balance disturbances.

Dysarthria, reflecting impaired speech motor coordination, is also observed in patients with SCD and MSA. It creates barriers to expressing the patient’s intentions and communicating with others, significantly impacting not only communication as a component of Activities of Daily Living (ADL) but also the quality of life of the patients and their families (3). According to Darley et al. (4), who investigated the auditory-perceptual characteristics of dysarthria in patients with neuromuscular diseases, patients with cerebellar ataxia exhibit motor inaccuracy, slowness, and hypotonia during speech production. These deficits result in articulatory, prosodic, and phonatory-prosodic inaccuracies, which are generally described as “scanning,” “explosive,” and “slurred.” Acoustic evaluations have revealed reduced speech rate, variability in speech rhythm, prolonged syllable duration, and flattened or excessive stress (5). Furthermore, it has been suggested that impaired regulation of expiratory pressure due to cerebellar ataxia may contribute to phonatory dysfunction (6).

Schalling et al. (7), in a perceptual study of dysarthria in patients with SCAs, noted that dysarthria is associated with articulatory inaccuracy, prosodic insufficiency, and phonatory insufficiency due to dyscoordination of speech and hypertonia and hypomotor activity of the larynx. Comparative acoustic analyses with healthy controls have similarly demonstrated reduced speech rate, lower alternating and sequential motion rates (AMR/SMR), and prolonged syllable and pause durations. Increased variability of segment durations and vocal instability, such as pitch variability in sustained phonation, were also observed. Additionally, regarding dysarthria in Multiple System Atrophy (MSA), an acoustic analysis by Rusz et al. (8) highlighted voice perturbations, slow and variable alternating motion rates (AMR), pitch fluctuations, excess intensity variations, prolonged phonemes, vocal tremor, and strained-strangled voice quality.

Thus, while dysarthria in patients with SCD and MSA resembles ataxic dysarthria, it has been noted that elements of hypokinetic and spastic dysarthria coexist because the pathology extends to the spinal cord and basal ganglia. Therefore, it is essential to evaluate the disease-specific characteristics of dysarthria in SCD and MSA. However, although auditory-perceptual and acoustic studies exist, no reports have described the characteristics of dysarthria in these patients from a functional anatomical perspective focusing on the dysfunction of individual speech organs.

Dysarthria assessments, including the Mayo Clinic System (4), speech intelligibility, and the Radboud Dysarthria Assessment (9), Frenchay Dysarthria Assessment (FDA) (10) and Frenchay Dysarthria Assessment Second Edition (FDA-2) (11) are based on perceptual assessment. One of them, FDA-2, is a comprehensive assessment instrument that observes dysarthria in a total of 26 items: reflexes, respiration, lips, palate, laryngeal, tongue, and intelligibility. FDA-2 requires no special equipment and can be administered in a clinical setting within 30 min. Enderby, who examined ataxic dysarthria in patients with cerebellar disease using the FDA-2, found decreased scores on the laryngeal and tongue items in speech, indicating the involvement of laryngeal and tongue dyscoordination (11). Hijikata et al. (12) evaluated dysarthria in several neurodegenerative diseases using the FDA-2, and they reported that patients with SCD and MSA had higher scores on the lip items compared to patients with cerebrovascular disease and Parkinson’s disease. Specifically, because cerebellar pathology impairs timing control, motor programming, and the execution of motor acts, we hypothesized that the resulting deficits in timing, pitch, and loudness control, and the incoordination of speech organs, are reflected in the respective items of the FDA-2. However, as far as the author could find, no studies have evaluated dysarthria in SCD and MSA both longitudinally and cross-sectionally, nor have they examined functional abnormalities and temporal changes in the various speech-related organs.

Clarification of the characteristics of dysarthria in patients with SCD and MSA would be useful for early diagnosis of dysarthria in patients with this disease and for determining the effectiveness of speech therapy. Therefore, the purpose of this study is to evaluate the dysarthria of patients with SCD and MSA cross-sectionally and longitudinally using the FDA-2 and to clarify the distinctive characteristics and progression patterns of dysarthria over time.

## 2 Materials and methods

This study is a single-center, retrospective cohort study.

### 2.1 Participants

The subjects were patients with SCD and MSA who were seen at least twice at the Department of Rehabilitation Medicine of National Hospital Organization Higashisaitama Hospital at intervals of at least 12 months from April 1, 2018 to October 31, 2023. For patients who had three or more visits during the observation period, only the first and last evaluations were included in the analysis. Patient demographics (age, gender, disease type, and disease duration) as well as the following clinical evaluation results were retrospectively collected from medical records.

### 2.2 Evaluation of dysarthria and clinical symptoms

In this study, dysarthria was assessed by the Japanese version of Frenchay Dysarthria Assessment Second Edition (FDA-2) by Hijikata et al. (12) and the Scale for the Assessment and Rating of Ataxia (SARA) language impairment. The FDA-2 consists of 26 items: reflexes (3 items), respiration (2 items), lips (5 items), palate (4 items), laryngeal (4 items), tongue (6 items), and intelligibility (3 items). These are assessed through examiner observation, structured tasks, and questions presented to the subject.

Each item is graded on a five-point scale from “a” (normal for age), “b” (mild abnormality noticeable to skilled observer), “c” (abnormality obvious but can perform task/movements with reasonable approximation), “d” (some production of task but poor in quality, unable to sustain, inaccurate or extremely labored), and “e” (unable to undertake task/movement/sound). Half-point scores are allowed if the subject’s performance falls between two adjacent levels. For example, if the patient’s behavior is a little better than Descriptor c but not as good as Descriptor b, make a mark between the two. These qualitative ratings are converted to a nine-point scale for analysis, with “e” corresponding to 0 and “a” corresponding to 4, resulting in a total score range from 0–104. Higher scores indicate better articulatory function. The FDA-2 has established content validity in its original form and demonstrated structural validity in the Japanese version used in this study. In addition, the Japanese version of FDA-2 validation study reported an inter-rater reliability (ICC) of 0.76 for total scores, indicating robust reliability (12). The item-wise distribution of scores provides each patient’s dysarthria characteristics, and repeated evaluations allow assessment of longitudinal changes. The Japanese version of the FDA-2 was administered by one of five experienced speech-language therapists (SLTs) at our hospital who had previously participated in the development and validation of the Japanese version of FDA-2. All of them met the competency requirements specified in the original FDA-2 publication, were experienced SLTs specializing in neuromuscular disorders, and had completed the standardized 120-min FDA-2 training program. However, the same SLT did not necessarily evaluate each patient at both time points. In addition, the speech subscale of SARA was used as a supplementary assessment of dysarthria. SARA is a scale for cerebellar ataxia (13), which includes eight domains: gait, stance, sitting, speech, finger chase, nose-finger test, fast alternating hand movements, and heel-shin test. SARA item 4 (SARA speech) is rated on a 7-point scale from 0 (normal) to 6 (unintelligible speech or anarthria). SARA item 1 (SARA gait) was also used to assess ataxic motor impairments. The item is scored from 0 (normal, able to take more than 10 steps without difficulty in walking, changing direction, and walking on one’s feet) to 8 (unable to walk even with assistance). Additionally, motor function was evaluated using the motor subscale of the Functional Independence Measure (FIM) (14, 15). SARA speech, SARA gait, and FIM motor scores were rated by one rehabilitation physician. The FDA-2 examiners were blinded to the SARA and FIM scores.

### 2.3 Statistical analysis

Statistical analyses were performed using SPSS version 29.0 (IBM Corp., Armonk, NY, USA).

As a cross-sectional study, correlations between the total FDA-2 and other clinical rating scales (SARA speech, SARA gait, and FIM motor items) at each session were analyzed using Spearman’s rank correlation coefficient.

As a longitudinal study, normal distribution was assessed using Shapiro–Wilk tests (*p* > 0.05). Variables with normal distributions (the results of the FDA-2 total score, respiration, lips, and palate item) at the first and last evaluations were evaluated with a paired t-test, and variables without normal distributions (the results of FDA-2 reflex, laryngeal, tongue, and intelligibility items, SARA speech, SARA gait, and FIM motor scores) were evaluated with Wilcoxon’s signed-rank test.

To identify factors contributing to FDA-2 intelligibility, multiple regression analysis was conducted with the first FDA-2 intelligibility as the dependent variable and the mean values of the FDA-2 respiration, lips, palate, laryngeal, and tongue items in the first evaluation as explanatory variables. Given the exploratory nature of this study and the need to identify the most parsimonious model from multiple potential predictors, a stepwise selection method was employed using the Akaike Information Criterion (AIC) for model selection. This approach allows for systematic evaluation of predictor combinations while balancing model fit and complexity, making it particularly suitable for identifying key contributing factors when theoretical guidance for variable selection is limited. Before the analysis, several assumptions were examined. Multicollinearity among the predictors was assessed using the variance inflation factor (VIF), with a VIF value ≥10 indicating multicollinearity. The assumptions of linearity, homoscedasticity, and normality were also examined. The assumption of linearity was confirmed by visually inspecting the residual plots, while homoscedasticity was verified by examining the scatter plots of standardized residuals against predicted values. The normality of the residuals was assessed using a Q-Q plot and a histogram. Additionally, influential observations were identified using standardized residuals (with absolute values >3 indicating potential outliers). The Durbin-Watson test was conducted to assess the independence of residuals, with values between 1.5 and 2.5 indicating no autocorrelation. This study was conducted with the approval of the Ethics Committee of the National Hospital Organization Higashisaitama Hospital (No. 23–1) and complies with the Declaration of Helsinki. A summary of the study was made publicly available, and all participants were allowed to opt out through our hospital website. All data were anonymized to protect participant confidentiality, and participants were informed of their right to withdraw at any time without affecting their medical care.

## 3 Results

A total of 22 patients with SCD and MSA who visited our department at least twice during the study period at intervals of at least 12 months were included. As one participant was unable to complete the full assessment, we excluded this individual from the statistical analysis. A total of 21 patients (7 females and 14 males) with a mean age of 57.4 ± 8.5 years were evaluated (Table 1 and Supplementary material). The disease types were SCA2 in 1, SCA3 in 8, SCA31 in 1, MSA-C in 8, and an undetermined disease type in 3. Undetermined cases were those who met the diagnostic criteria for probable SCD but were diagnosed by our neurologist as an undetermined disease type when genetic testing was negative for SCA1, SCA2, SCA3, SCA6, SCA7, SCA17, and DRPLA. The median number of years since onset was 7 years, and the mean interval between the first and last examinations was 31.1 ± 15.6 months.

**Table 1:** Baseline clinical characteristics.

Characteristic	Patients (*n* = 21)	
Male, *n* (%)	14 (66.7)	
Age, mean (SD)	57.4 (8.5)	
Diagnosis, *n*	SCA2	1
	SCA3	8
	SCA31	1
	MSA-C	8
	undetermined	3
Years since onset [median(IQR)]	7.00 (4.00–19.00)	
Evaluation interval, months (mean(SD), months)	31.1 (15.6)	

SARA speech, SARA gait, and FIM motor items at the first and final evaluations are shown in Table 2. Wilcoxon’s signed-rank test showed significant differences between the initial and final evaluations for all of these.

**Table 2:** Clinical measures and the results Wilcoxon’s signed-rank test.

Variable	The first evaluation [median (IQR)]	The last evaluation [median (IQR)]	*Z*-value	*p*-value	Effect size(r)
SARA speech	2 (2–2)	2 (2–3)	−2.460	0.014	−0.537
SARA gait	5 (5–7)	8 (6.5–8)	−3.581	<0.001	−0.781
FIM motor item	76 (64–81)	55 (32–75)	−3.643	<0.001	−0.795

^1^SARA, the Scale for the Assessment and Rating of Ataxia.

^2^Data as median (IQR); analyzed by Wilcoxon’s signed-rank test; Effect size: *r* = Z/√N; *p < 0.05.

**Figure 1:**
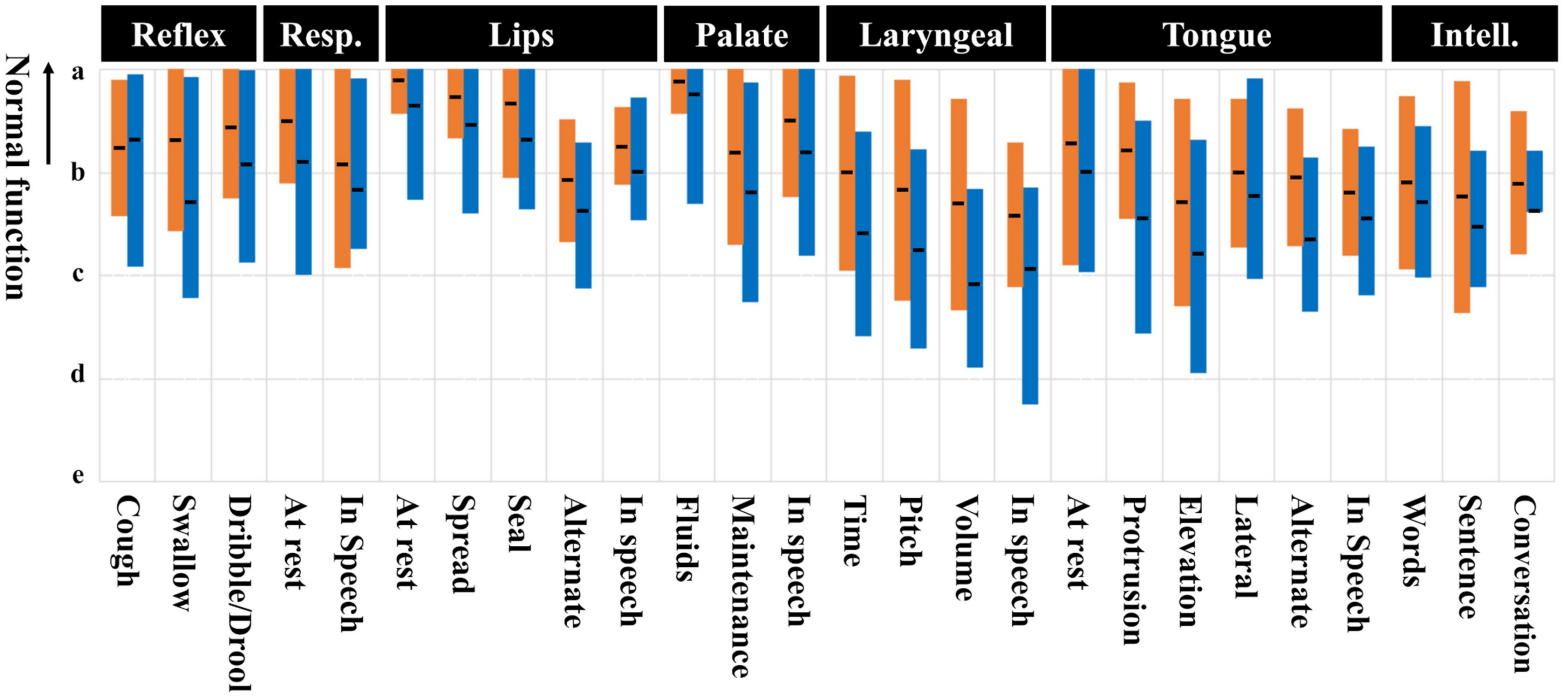
FDA-2 item scores. Orange bars represent the first evaluation and blue bars represent the last evaluation. Black lines indicate the mean, and the colored areas indicate the standard deviation.

Table 3 and Figure 1 present the FDA-2 results for the first and final evaluations. As shown in Figure 1, scores for lip, palate, laryngeal, and tongue items were generally lower during speech-related and dynamic tasks (e.g., “in speech,” “alternate”) than during rest or static tasks (e.g., “at rest,” “spread”). This pattern suggests that dyscoordination may underlie the observed impairments. Table 3 also shows that there was a significant difference between the first and final total scores, with a significant decrease in scores for the reflexes, lips, laryngeal, and tongue items in the major items, but no significant change in the respiration, palate, and intelligibility items.

**Table 3:** FDA-2 section scores.

Variable	The first evaluation	The last evaluation	Difference	95%CI	Statistics	Effect size	*p*-value
Total score (0–104)^1^	81.81 ± 11.02	72.52 ± 11.59	−9.29 ± 8.71	−13.25–−5.32	*t* = 4.887	−3.148	<0.001*
Reflex (0–12)^1^	9.98 ± 1.68	9.23 ± 1.66	−0.74 ± 1.62	−1.47–0.00	*t* = 2.091	−0.581	0.049*
Respiration (0–8)^1^	6.55 ± 1.39	6.02 ± 1.66	−0.52 ± 1.41	−1.17–0.12	*t* = 1.703	−0.438	0.104
Lips (0–20)^1^	17.33 ± 1.81	15.74 ± 2.68	−1.60 ± 2.46	−2.71–−0.48	*t* = 2.974	−1.020	0.007*
Palate (0–12)^1^	10.43 ± 1.78	9.85 ± 1.90	−0.57 ± 1.77	−1.38–0.23	*t* = 1.480	−0.428	0.155
Laryngeal (0–16)^2^	11.50 (8.50–13.00)	9.00 (7.00–10.00)	-	-	*Z* = −2.924	*r* = −0.638	<0.001*
Tongue (0–24)^2^	18.00 (16.50–20.00)	16.00 (12.00–18.50)	-	-	*Z* = −2.953	*r* = −0.644	0.002*
Intelligibility (0–12)^2^	9.00 (7.50–9.75)	8.50 (6.75–9.00)	-	-	*Z* = −1.193	*r* = −0.260	0.229

^1^Data as mean ± SD; analyzed by paired *t*-test; Effect size: Cohen’s d.

^2^Data as median (IQR); analyzed by Wilcoxon’s signed-rank test; Effect size: *r* = Z/√N; *p < 0.05.

Table 4 shows that significant correlations were found between the total FDA-2 scores at each assessment and all other clinical evaluation scales (initial SARA speech: *ρ* = −0.525, SARA gait: *ρ* = −0.681, FIM motor items: *ρ* = 0.714, final SARA speech: *ρ* = −0.627, SARA gait: *ρ* = −0.700, FIM motor items: *ρ* = 0.804) (*p* < 0.05).

**Table 4:** Spearman’s rank correlation coefficient between FDA-2 and clinical measures.

Variables	The first evaluation	Effct size (*ρ*^2^)	The last evaluation	Effect size(*ρ*^2^)
SARA speech	*ρ* = −0.525*	0.276	*ρ* = −0.627*	0.393
SARA gait	*ρ* = −0.681*	0.464	*ρ* = −0.700*	0.490
FIM	*ρ* = −0.714*	0.510	*ρ* = −0.804*	0.646

SARA, the Scale for the Assessment and Rating of Ataxia; *p < 0.05.

Table 5 shows the results of a multiple regression analysis with the first FDA-2 intelligibility as the dependent variable and the means of the first FDA-2 respiration, lips, palate, laryngeal, and tongue as explanatory variables. The explanatory variables were conducted using a stepwise method to ensure that multicollinearity did not occur (Variance inflation factor: VIF < 10). A significant relationship was found between initial FDA-2 intelligibility and FDA-2 laryngeal items (Sβ = 0.766, *p* = 0.047, R^2^ = 0.416). The Durbin-Watson ratio was 1.53, and no outliers with a difference between predicted and residual values exceeding ±3 were observed.

**Table 5:** Multiple regression analysis for the first FDA-2 intelligibility.

Variable	B	95% CI	Sβ	SE	*t*-value	*p*-value
Respiration	1.207	−0.654–3.069	0.371	0.873	1.383	0.187
Lips	−0.259	−3.462–2.945	−0.041	1.503	−0.172	0.866
Palate	−0.818	−3.631–1.995	−0.216	1.32	−0.620	0.545
Laryngeal	2.279	0.038–4.520	0.766	1.051	2.168	0.047*
Tongue	−1.698	−4.453–1.058	−0.366	1.293	−1.313	0.209
Adjusted R^2^	0.416					

*p < 0.05.

## 4 Discussion

### 4.1 Strength of this study

This study is the first to conduct cross-sectional and longitudinal evaluations of dysfunction in individual speech-related structures in patients with SCD and MSA, using the FDA-2. SCD and MSA are progressive neurodegenerative diseases, exacerbating ataxic symptoms and other neurological clinical syndromes as the disease progresses, leading to further ADL decline (16).

Although dysarthria in SCD and MSA have traditionally been expected to resemble ataxic dysarthria observed in other cerebellar disorders, the involvement of additional structures such as the spinal cord and basal ganglia results in distinct dysarthric features. While some perceptual studies of dysarthria in SCD and MSA have been reported, no previous studies have systematically analyzed the disorder in terms of functional abnormalities in individual speech-related organs.

Therefore, we believe this study, which used the FDA-2 to evaluate dysarthria in patients with SCD and MSA both longitudinally and cross-sectionally, provides meaningful insights and contributes significantly to our understanding of the pathophysiology and clinical progression of dysarthria in this population.

### 4.2 Progression of dysarthria

In the present study, significant correlations were found between the FDA-2 total score and both FIM motor items and SARA gait in patients with SCD and MSA. In the last evaluation, which was conducted more than one year after the first one, the FDA-2 total was significantly lower than in the first evaluation, indicating that dysarthria in SCD progressively worsens over time in parallel with overall motor deterioration. These findings are consistent with previous reports (3).

### 4.3 Characteristics of each speech organ and longitudinal change

In the cross-sectional evaluation of this study, the scores for lips, palate, tongue, and laryngeal items were generally lower during speech-related and dynamic tasks compared to static or rest conditions, in both the first and last evaluations. These patterns are consistent with previous reports indicating that temporal and spatial dysregulation of articulatory structures is a core mechanism underlying ataxic dysarthria (17). Therefore, the findings of this study provide objective support for the presence of articulatory dyscoordination in patients with SCD and MSA, particularly in tasks involving dynamic, volitional, or speech-specific movement.

In the evaluation of each function, the scores for the lips and palate were generally higher, while the scores for the laryngeal and tongue tended to be lower. In a report by Eigentler et al. (18) using the FDA to evaluate dysarthria in patients with Friedrich’s disease, voice was the most impaired, followed by palate and tongue with lower scores, and lips, jaw, and respiration were only mildly impaired. Although the FDA and the FDA-2 differ in some of the endpoints, the findings indicate that laryngeal function was most severely impaired. In contrast, while the palate and tongue scores were similar in Friedrich’s disease, our study found that palatal function was relatively better preserved. The dysarthria in Friedrich’s disease has been reported to be characterized by palatopharyngeal dysfunction (19), suggesting that dysarthria in SCD/MSA and Friedrich disease may have different features, even in diseases that also present with ataxic dysarthria.

Regarding the lips, static items such as “at rest,” “spread,” and “seal” scored higher than the dynamic repetitive movements, “alternate” and “in speech.” Patients with SCD and MSA have cerebellar ataxia, resulting in loss of velocity control of lip movements and disorganized timing of movement initiation (13). It can be inferred that the scores of the static assessment of “at rest,” “spread,” and “seal” were higher than those of “alternate” and “in speech,” which assess dynamic repetitive movements. The current study also showed a decrease in scores over time. In a report of a similar disease, Borel et al. (20) examined the association between facial muscle movements and dysarthria in Friedrich’s disease and reported no progression of lip movement dysfunction over a 1-year observation period. The mean examination interval in this study was 31.1 ± 15.6 months, which is longer than that reported by Borel et al. (20) and may have revealed a slow, progressive functional decline. These results suggest that the functional decline in dysarthria of SCD and MSA patients may be gradual, with a decrease in scores during repetitive movement tasks and conversation compared to the resting state due to dyscoordination in lip movements.

The palate was the only speech organ that did not show a significant decline over time. This aligns with previous reports that velopharyngeal dysfunction, while occasionally presenting as hypernasality in SCA, is generally less prominent in ataxic dysarthria (4). In patients with neuromuscular diseases, velopharyngeal dysfunction during phonation may precede that during swallowing (21), and it has been reported that dysphagia may develop following the onset of dysarthria in SCA patients (22). Therefore, in the present study as well, although the scores for the palate were higher than those for other speech organs, the “maintenance” and “in speech” items, which assess velopharyngeal function during phonation, showed lower scores compared to the “fluids” item, which assesses function during swallowing. These findings suggest that palatal function tends to be relatively preserved in the dysarthria of this disease, and that velopharyngeal insufficiency during swallowing may appear later than that during phonation.

Compared to the lip and palate items, the laryngeal and tongue items showed lower score distributions. In the present study, laryngeal scores were generally low, with the largest discrepancy between scores for rest/static movement tasks and repetitive movement tasks. This pattern aligns with previous reports of laryngeal regulation disorders associated with cerebellar degeneration-induced feedback dysfunction in dysarthria of SCD and MSA patients (23). Schalling et al. (7) have pointed out that laryngeal hypertonia can be associated with vocal impairments, such as harshness, strained-strangled vocal quality, and glottal fry. Furthermore, in the multiple regression analysis using the first FDA-2 intelligibility as the dependent variable, the larynx was the only significant predictor. While ataxic dysarthria tends to maintain higher speech intelligibility compared to other dysarthria types (24), these findings suggest that laryngeal dysfunction may be particularly associated with reduced speech intelligibility in SCD and MSA patients. However, the observed R^2^ of 0.416, while suggesting a meaningful association between laryngeal function and intelligibility, should be considered preliminary given the exploratory nature of this analysis and the limited sample size. Furthermore, as the FDA-2 intelligibility relies on perceptual evaluation without acoustic analysis or visual functional assessment, future studies should incorporate instrumental measures, including acoustic analysis, visual evaluation of speech organ function, and correlation with pathological changes, to provide a more comprehensive understanding of dysarthria progression in these populations.

The tongue item also showed generally low score distributions, with movement tasks being lower than at rest. This pattern is consistent with the incoordination typically seen in cerebellar degeneration. However, lesions in SCD and MSA often extend to the spinal cord and brainstem, distinguishing them from other cerebellar degenerative diseases. Previous studies have reported that the medulla extends axonal projections to both hypoglossal nerves, and it has been speculated that medullary atrophy in SCD and MSA patients may be associated with impaired tongue movements and, consequently, dysarthria (6). Furthermore, MSA pathology also involves the basal ganglia. These findings and anatomical considerations may potentially suggest that tongue dysfunction in SCD and MSA patients involves multiple pathological processes distinct from those seen in typical ataxic dysarthria. However, the relative contributions of these different pathological processes to tongue dysfunction remain unclear and warrant systematic investigation using instrumental assessment methods.

In ataxic dysarthria, impaired control of expiratory pressure due to cerebellar incoordination has been suggested as a contributing factor. In patients with SCD and MSA, dysarthria may be influenced not only by such cerebellar coordination-related respiratory dysfunction but also potentially by central respiratory impairment caused by medullary lesions (6). Since in the FDA-2 respiratory items, “at rest” evaluates the smoothness of deep exhalation, and “in speech” assesses the number of breaths required to count from 1 to 20 aloud, bothtasks reflect impairments in vital capacity and expiratory pressure regulation, resulting in lower scores in this study. However, few previous studies have reported on the relationship between respiratory dysfunction and dysarthria in patients with SCD or MSA. Furthermore, to the best of our knowledge, no studies have explored this relationship using objective physiological assessments such as spirometry. Therefore, further research is warranted to clarify the association between respiratory function and dysarthria in patients with SCD and MSA.

In conclusion, this study aimed to investigate the characteristics of dysarthria in patients with SCD and MSA by conducting both cross-sectional and longitudinal assessments using the Frenchay Dysarthria Assessment Second Edition (FDA-2), which allows a comprehensive evaluation of functional impairments in speech-related organs. The results indicated that dysarthria progresses in parallel with disease progression in SCD and MSA patients, with cerebellar incoordination suspected to contribute to dysfunction in various speech subsystems. Notably, while laryngeal function tended to be impaired from an early stage, palatal function appeared to be relatively preserved, suggesting the potential for early detection of dysarthria through laryngeal function assessment. Furthermore, by utilizing the FDA-2 to evaluate functional changes in each speech organ both cross-sectionally and over time, it may be possible to objectively and precisely monitor the progression of dysarthria in SCD and MSA patients. This approach may facilitate the selection of appropriate rehabilitation targets in speech-language therapy and enable more accurate evaluation of treatment outcomes.

## 5 Limitation

This study has several limitations. First, the sample size was small, and patients with multiple subtypes were included. SCAs comprise more than 40 subtypes, each with distinct characteristics, and the disease progression rate also varies among subtypes—for instance, MSA typically progresses more rapidly than most SCAs. We acknowledge that the lack of individual assessment of natural history and detailed dysarthric characteristics in our study population represents a significant limitation. In addition, although this study included patients with a median disease duration of 7 years, differences in disease progression speed by subtype mean that heterogeneity in disease type and duration must be taken into account when interpreting the results. Therefore, the present findings may not comprehensively represent the full range of dysarthria characteristics across all SCD and MSA patients. Second, multiple paired comparisons were performed for each FDA-2 subscore without correction for multiple comparisons. As the purpose of this analysis was to explore how each speech-related organ changes over time rather than to test a single overarching hypothesis, we did not apply a correction for multiple testing. However, this approach may increase the risk of Type I error, and therefore, the results of the subitem analyses should be interpreted as exploratory. In addition, the multiple regression model may be underpowered, as five predictors were initially included with only 21 observations, raising concerns about overfitting and unstable parameter estimates. Given the borderline significance of the final model (*p* = 0.047), the results of the regression analysis should also be interpreted as exploratory and with caution. Third, although the FDA-2 has demonstrated adequate validity in previous studies (12) and trained SLTs performed assessments, the same SLT did not necessarily evaluate each patient at both time points. No intra-rater or inter-rater agreement was formally calculated in this sample, and this should be considered when interpreting the results. Moreover, as this was a retrospective study, information regarding speech-language therapy during the observation period was not consistently documented for all patients, which may have influenced the longitudinal changes in the FDA-2 scores. Fourth, since the FDA-2 relies on perceputual evaluation without instrumental assessments such as acoustic analysis or detailed motor function evaluation of individual speech-related organs, this may potentially result in floor or ceiling effects. In this regard, the apparent stability of palate scores over time could also reflect limited sensitivity of the FDA-2 palate items and possible floor or ceiling effects, in addition to true relative preservation of palatal function. Looking forward, further studies incorporating instrumental assessments, including acoustic analysis, visual functional evaluation, and correlation with pathological changes, are warranted to provide a more comprehensive understanding of dysarthria progression in these populations. For instance, Dubbioso et al. (25) demonstrated the potential utility of integrating quantitative voice analysis and digital technology for early diagnosis and longitudinal evaluation of dysarthria in progressive neuromuscular diseases. Finally, because these are rare diseases, single-center studies inherently face limitations in sample size and diversity. Additionally, although this is the first longitudinal study to evaluate dysarthria in SCD and MSA patients using the FDA-2, assessments were conducted at only two time points, separated by more than 12 months. Future research should involve longer-term follow-up and evaluations at three or more time points to more precisely and sensitively capture the temporal changes in dysarthria.

## 6 Conclusion

Dysarthria in patients with spinocerebellar degeneration (SCD) and multiple system atrophy (MSA) was investigated using the Frenchay Dysarthria Assessment Second Edition (FDA-2) through both cross-sectional and longitudinal analyses. A correlation was observed between the FDA-2 total scores and other clinical indicators, and the longitudinal assessment revealed a decline in the FDA-2 total scores over time, suggesting that dysarthria in SCD and MSA worsens with disease progression. The FDA-2 scores for lips, palate, tongue, and laryngeal items were generally lower in tasks involving dynamic, volitional, or speech-specific movement compared to those at rest, which may reflect cerebellar contributions to temporal precision and scaling. Additionally, the FDA-2 laryngeal and tongue items tended to decline, while palatal function appeared to be relatively preserved. Exploratory multiple regression analysis using the first FDA-2 intelligibility scores as the dependent variable suggested that laryngeal function may contribute to the reduction in intelligibility. As this study included a small number of patients with various disease subtypes, and the FDA-2 relies on perceptual evaluation without instrumental assessments, further detailed investigation is warranted.

## Data Availability

The raw data supporting the conclusions of this article will be made available by the authors, without undue reservation.
