# Supplementary material for: Characteristics of dysarthria in patients with spinocerebellar degeneration and multiple system atrophy: a cross-sectional and longitudinal study using the Frenchay Dysarthria Assessment Second Edition (FDA-2)
Source: Front Neurol. 2026 Jan 7;16:1734012. doi: 10.3389/fneur.2025.1734012 (PMC12819311; doi:10.3389/fneur.2025.1734012)
Supplement: Supplementary file 1 [file Table_1.docx]

| Supplementary TABLE 1. Demographics of study population | | | | | |
| --- | --- | --- | --- | --- | --- |
| **Diagnosis** | **SCA2(n=1)** | **SCA3(n=8)** | **SCA31(n=1)** | **MSA-C(n=8)** | **undetermined(n=3)** |
| Male,n(%) | 1 (100.0) | 5 (62.5) | 1 (100.0) | 4.0 (50.0) | 3 (100.0) |
| Age,mean(SD) | 64.0 | 52.9 (9.6) | 75.0 | 60.4 (3.1) | 53.7 (7.2) |
| Years since onset (median(IQR)) | 35.0 | 13.0 (7.3-19.8) | 17.0 | 4.00 (2.3-5.8) | 19.0 (5.0-19.0) |
| Evaluation interval (mean(SD), months) | 28.0 | 37.4 (14.2) | 12.0 | 24.4(11.7) | 21.7 (7.2) |
